# Pharmacological activation of CB_2_ receptors counteracts the deleterious effect of ethanol on cell proliferation in the main neurogenic zones of the adult rat brain

**DOI:** 10.3389/fncel.2015.00379

**Published:** 2015-09-29

**Authors:** Patricia Rivera, Eduardo Blanco, Laura Bindila, Francisco Alen, Antonio Vargas, Leticia Rubio, Francisco J. Pavón, Antonia Serrano, Beat Lutz, Fernando Rodríguez de Fonseca, Juan Suárez

**Affiliations:** ^1^UGC Salud Mental, Instituto de Investigación Biomédica de Málaga, Universidad de Málaga-Hospital Universitario Regional de MálagaMálaga, Spain; ^2^Departament de Pedagogia i Psicologia, Facultat de Ciències de l'Educació, Universitat de LleidaLleida, Spain; ^3^Institute of Physiological Chemistry, University Medical Center of the Johannes Gutenberg-University of MainzMainz, Germany; ^4^Departamento de Anatomía y Medicina Legal, Universidad de MálagaMálaga, Spain

**Keywords:** alcohol, ACEA, JWH133, CB_1_ receptor, CB_2_ receptor, neurogenesis

## Abstract

Chronic alcohol exposure reduces endocannabinoid activity and disrupts adult neurogenesis in rodents, which results in structural and functional alterations. Cannabinoid receptor agonists promote adult neural progenitor cell (NPC) proliferation. We evaluated the protective effects of the selective CB_1_ receptor agonist ACEA, the selective CB_2_ receptor agonist JWH133 and the fatty-acid amide-hydrolase (FAAH) inhibitor URB597, which enhances endocannabinoid receptor activity, on NPC proliferation in rats with forced consumption of ethanol (10%) or sucrose liquid diets for 2 weeks. We performed immunohistochemical and stereological analyses of cells expressing the mitotic phosphorylation of histone-3 (phospho-H3+) and the replicating cell DNA marker 5-bromo-2'-deoxyuridine (BrdU+) in the main neurogenic zones of adult brain: subgranular zone of dentate gyrus (SGZ), subventricular zone of lateral ventricles (SVZ) and hypothalamus. Animals were allowed *ad libitum* ethanol intake (7.3 ± 1.1 g/kg/day) after a controlled isocaloric pair-feeding period of sucrose and alcoholic diets. Alcohol intake reduced the number of BrdU+ cells in SGZ, SVZ, and hypothalamus. The treatments (URB597, ACEA, JWH133) exerted a differential increase in alcohol consumption over time, but JWH133 specifically counteracted the deleterious effect of ethanol on NPC proliferation in the SVZ and SGZ, and ACEA reversed this effect in the SGZ only. JWH133 also induced an increased number of BrdU+ cells expressing neuron-specific β3-tubulin in the SVZ and SGZ. These results indicated that the specific activation of CB_2_ receptors rescued alcohol-induced impaired NPC proliferation, which is a potential clinical interest for the risk of neural damage in alcohol dependence.

## Introduction

The excessive consumption of alcohol is associated with alterations in brain physiology, structure and function. Alcohol-induced neurodegeneration and cognitive deficits occur due to neuronal cell death and the loss of neurogenesis in acute and chronic alcohol intoxication (Nixon and Crews, 2004). Consequently, individuals with alcohol dependence exhibit significant volume loss in cortical and subcortical brain structures, including gray and white matter shrinkage (White, 2003; Crews and Nixon, 2009). A thinning of the granular layer of the dentate gyrus in animal models of alcohol exposure is attributed to neuronal loss and a decrease in newborn cells, which contributes to impairments in hippocampus-dependent tasks (Santín et al., 2000; Roberto et al., 2002; Herrera et al., 2003; Shors et al., 2012). The mechanisms underlying alcohol-induced neuronal damage and death are complex and not well understood. Proposed explanations include excitotoxicity associated with excessive neurotransmitter release and oxidative stress. The endocannabinoid system in the most sensitive corticolimbic brain regions, such as the hippocampus, tightly regulates these processes (Crews et al., 2004; Katona and Freund, 2012).

The endocannabinoid system is a growing interest because it mediates neuroprotective effects after damage, and it affects neural stem cell proliferation (Stella, 2010; Katona and Freund, 2012; Galve-Roperh et al., 2013; Bandiera et al., 2014). The activation of the cannabinoid receptors (CB_1_ and CB_2_ receptors) and the inhibition of the *N*-acylethanolamides (NAEs) hydrolyzing enzyme FAAH promote neural proliferation, which was observed in aged mouse brains (Aguado et al., 2005; Goncalves et al., 2008). Therefore, CB_1_ receptor activation promotes neuronal differentiation and maturation and protects neurons from brain injury (Parmentier-Batteur et al., 2002; Marsicano et al., 2003; Fowler et al., 2010; Compagnucci et al., 2013). Selective CB_2_ receptor stimulation prevents deficits in neurogenesis, microglial activation and cognitive impairment, including the undesired psychoactive effects of neuronal CB_1_ receptor activation (Ashton and Glass, 2007; Goncalves et al., 2008; Stella, 2010; Palazuelos et al., 2012; Rom and Persidsky, 2013; Avraham et al., 2014).

Most studies suggested reduced brain cannabinoid activity during alcohol consumption, likely from the low expression levels of the CB_1_ and CB_2_ receptors (Basavarajappa, 2007; Ishiguro et al., 2007; Serrano et al., 2012). The impairment of neurogenesis during alcohol intoxication may be associated with a reduced cannabinoid activity (Ortiz et al., 2004; Ferrer et al., 2007; Crews and Nixon, 2009; Rubio et al., 2009), and the potential positive modulation of neurogenic responses promoted by the exogenous administration of cannabinoid receptor agonists may be beneficial in alcoholism. However, one of the major concerns against the use of this class of compounds arose from results that demonstrated that the CB_1_ receptor agonists CP 55,940 and WIN 55,212-2, which bind both CB_1_ and CB_2_ receptors with similar affinities, may increase voluntary alcohol consumption and alcohol drinking motivation, which were specifically observed in Sardinian alcohol-preferring (sP) rats (Hungund et al., 2003; Colombo et al., 2005). In contrast, other studies indicated that novel and selective agonists for CB_1_ or CB_2_ receptors may exhibit beneficial effects in alcoholism, such as anti-oxidant activity in the liver (Tsvetanova et al., 2006) or reduced alcohol intake and preference (Vasiljevik et al., 2013; Al Mansouri et al., 2014). Discrepancies also exist for modulators of endocannabinoid tone when these agents were administered in distinct experimental models of alcohol exposure (Serrano and Parsons, 2011). For example, administration of the FAAH inhibitor URB597 into the prefrontal cortex increased alcohol intake in rodents (Basavarajappa et al., 2006; Hansson et al., 2007; Vinod et al., 2008). However, the intraperitoneal administration of URB597 did not show an alcohol abuse risk, and it reduced anxiety associated to alcohol withdrawal in Wistar and sP rats (Cippitelli et al., 2008). The AEA transport inhibitor AM404 reduced alcohol self-administration (Cippitelli et al., 2007). However, whether the selective activation of cannabinoid CB_1_ and CB_2_ receptors or the pharmacological elevation of endocannabinoid tone counteract cell proliferation impairment in the adult brain of rats exposed to alcohol is not known.

The present study designed an experimental approach to investigate the protective effects of the repeated administration (5 days) of the FAAH inhibitor URB597 (at an effective dose of 0.3 mg/kg; Piomelli et al., 2006), ACEA (3 mg/kg) and JWH133 (0.2 mg/kg) on brain structures that display NPC proliferation in alcohol-exposed rats. Immunohistochemistry and stereology assessed cells that expressed the mitotic Ser-10 phosphorylation of histone-3 (phospho-H3+) and contained the replicating cell DNA marker 5-bromo-2′-deoxyuridine (BrdU+) in the main neurogenic zones [subgranular zone of dentate gyrus (SGZ), subventricular zone of lateral ventricles (SVZ) and hypothalamus] in adult rats with a forced consumption of ethanol (10%) and sucrose liquid diets for 2 weeks. Precursor and mature phenotyping of the BrdU+ cells was also evaluated.

## Materials and methods

### Ethics statement

The Ethics and Research Committee at the Universidad de Málaga approved the protocols for animal care and use. All experimental animal procedures were performed in strict accordance with the European Communities directive 86/609/ECC (24 November 1986) and Spanish legislation (BOE 252/34367-91, 2005) regulating animal research. All efforts were made to minimize animal suffering and reduce the number of animals used.

### Animals

Male Wistar rats (approximately 250 g, 10–12 weeks old; Charles Rivers, Barcelona, Spain) were housed individually in cages and maintained in standard conditions (Servicio de Estabulario, Facultad de Medicina, Universidad de Málaga) at 20 ± 2°C room temperature with 40 ± 5% relative humidity and a 12-h light/dark cycle with a dawn/dusk effect. Water and standard rodent chow (Prolab RMH 2500, 2.9 kcal/g) were available *ad libitum*, unless otherwise indicated for specific experimental procedures.

### Alcoholic diet habituation

Rats were first food-deprived to 95% of their free-feeding weight, and they were allowed *ad libitum* access to a complete and balanced chocolate-flavored liquid food (0.97 kcal/mL; Glucerna SR, Abbott Laboratories, Madrid, Spain) until a stable rate of feeding was reached (~50 mL/day, up to 2–3 days). Rats were divided into two groups (*n* = 24) and fed the chocolate-flavored liquid diet supplemented with 10% (*v/v*) ethanol (alcoholic liquid diet) or 14.7% (*w/v*) sucrose (sucrose liquid diet; Serrano et al., 2012). Alcoholic and sucrose liquid diets were formulated to contain the same calories (1.4 kcal/mL). The same volume of ethanol liquid diet consumed *ad libitum* was given the next day to the rats fed the sucrose liquid diet. The rats were maintained under a controlled-isocaloric pair feeding period until a stable rate of alcoholic consumption was reached (12.4 ± 1.4 g of ethanol/kg body weight/day, up to 1 week). Water was available *ad libitum* during experimentation. Cumulative intake and body weight gain were periodically monitored.

### Administrations of URB597, ACEA, and JWH133

**Figure 1:**
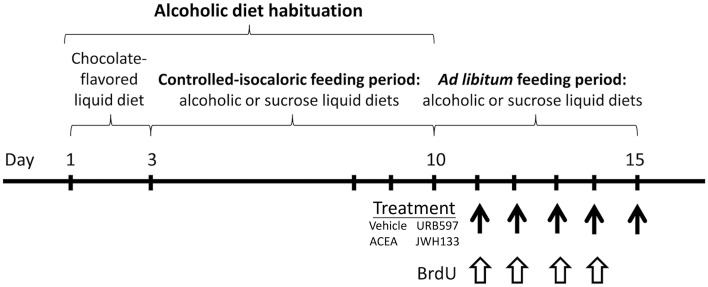
**Time line of the experimental design that summarize the periods of feeding and treatment used in the present study**.

Rats were fed *ad libitum* with both liquid diets (ethanol or sucrose) after alcoholic diet habituation. The following drugs were intraperitoneally administered: FAAH inhibitor URB597 (cyclohexyl carbamic acid 3′-carbamoyl-biphenyl-3-yl ester, IC_50_ = 3−5 nM; Cayman Chemical, cat. no. 10046, Ann Arbor, MI, USA), the potent and highly selective CB_1_ receptor agonist ACEA (arachidonyl-2′-chloroethylamide, K_*i*_ = 1.4 nM; Tocris, cat. no. 1319), and the potent and selective CB_2_ receptor agonist JWH133 (K_*i*_ = 3.4 nM; Tocris, cat. no. 1343). Drugs were dissolved in a vehicle containing 33% (*v*/*v*) DMSO in a sterile 0.9% NaCl solution and administered in a final volume of 1 mL/kg body weight. We selected the optimal dose at which treatment would be more effective in neuroprotection, as previously described (Piomelli et al., 2006; Goncalves et al., 2008; Galan-Rodriguez et al., 2009). URB597, ACEA and JWH133 were repeatedly administered at doses of 0.3, 3, and 0.2 mg/kg body weight, respectively, for 5 consecutive days at 24-h intervals (8:00 a.m.). The animals were sacrificed 2 h after the last drug injection. We obtained eight experimental groups depending on the diet and treatment (*n* = 6): vehicle-treated sucrose-fed group, URB597-treated sucrose-fed group, ACEA-treated sucrose-fed group, JWH133-treated sucrose-fed group, vehicle-treated ethanol-fed group, URB597-treated ethanol-fed group, ACEA-treated ethanol-fed group and JWH133-treated ethanol-fed group (Figure 1).

### BrdU administration

5′-bromo-2′-deoxyuridine (BrdU, cat. no. B5002, Sigma, St. Louis, MO, USA) was dissolved at 15 mg/mL in a sterile 0.9% NaCl solution. BrdU was administered intraperitoneally (i.p.) during the *ad libitum* feeding period at a dose of 50 mg/kg body weight twice daily at 10-h intervals (08:00, 18:00 h) for 4 consecutive days (Cifuentes et al., 2011).

### Sample collection

All animals were anesthetized (sodium pentobarbital, 50 mg/kg body weight, i.p.) 2 h after the last dose of treatment. Blood samples were transcardially collected into tubes containing EDTA-2Na (1 mg/mL blood) and centrifuged (1600 *g* for 10 min, 4°C). Plasma was frozen and stored at −80°C for biochemical analyses. Brains were frozen at -80°C and prepared on dry ice to obtain 1-mm thick sections using razor blades and a rat brain slicer matrix. The striatum, hippocampus and hypothalamus were precisely removed from 2.28 to −0.24 mm and −2.16 to −4.20 mm from Bregma levels (Paxinos and Watson, 2007) using fine surgical instruments. Brain samples were weighed and stored at −80°C until use in liquid chromatography—multiple reaction monitoring (LC-MRM) analyses. A replicated batch of rats were transcardially perfused with 4% formaldehyde in 0.1 M phosphate buffer (PB), and the brains were dissected and kept in the same fixative solution overnight at 4°C. These brains were cryoprotected and cut into 30-μm-thick coronal sections using a sliding microtome (Leica VT1000S). Sections were divided in eight parallel series until use for immunostaining.

### Anandamide quantification

We performed a quantitative analysis of plasma and brain anandamide (AEA) following a recently published protocol by Lomazzo et al. (2015) with slight modification to assess the inhibition of FAAH activity by URB597. Plasma endocannabinoid extract was resuspended in 50 μL (1:1, ACN:H_2_0), and an aliquot was diluted 1:5 (1:1, ACN:H_2_0) prior to injection in the LC/MRM. Aliquots (20 μL) were injected. AEA values were normalized to the starting plasma volume or the tissue weight used for extraction (see Supplementary Material).

### Biochemical and enzymatic analysis

The hepatic enzymes gamma-glutamyl transpeptidase (γGT), glutamate pyruvate transaminase (GPT) and glutamic oxaloacetic transaminase (GOT), and creatinine and bilirubin were analyzed using commercial kits according to the manufacturer's instructions in a Hitachi 737 Automatic Analyzer (Hitachi Ltd., Tokyo, Japan). A calibration curve and internal controls were included in each assay.

### Immunohistochemistry

Free-floating coronal sections from −2.16 to −4.20 Bregma levels (hippocampus and hypothalamus) and 2.28 to -0.24 Bregma levels (striatum) of each parallel series were selected for each immunohistochemistry (Rivera et al., 2015). Sections were incubated overnight in the following diluted primary antibodies at 4°C: rabbit anti-phospho-histone H3 (Ser-10; 2 μg/ml, Upstate, cat. no. 06-570) and mouse anti-BrdU (1:2000, Hybridoma Bank, Iowa City, IA, USA; ref. G3G4; Cifuentes et al., 2011; Rivera et al., 2011, 2013). Sections were incubated in the respective secondary antibody for 90 min: biotinylated goat anti-mouse IgG (1:500, Sigma; cat. no. B7264) or biotinylated donkey anti-rabbit IgG (1:500, Amersham, Little Chalfont, England; cat. no. RPN 1004). The sections were incubated in ExtrAvidin peroxidase (Sigma, St. Louis, MO) diluted 1:2000 in darkness at room temperature for 1 h. Immunolabeling was revealed with 0.05% diaminobenzidine (DAB; Sigma), 0.05% nickel ammonium sulfate and 0.03% H_2_O_2_ in PBS.

### Stereological cell quantification

The average density of immunoreactive cells per animal was quantified using stereological methods. Estimations of the number of cells per section (30 μm deep) and area (mm^2^) in both hemispheres was calculated according to the following formula: *N*_*a*_ = Σ(*Q*−)∕Σ(*a*_*str*_), where Σ*Q*- is the total number of positive cells counted per animal, and *a*_*str*_ is the area of the structure analyzed. Each structure analyzed consisted of approximately 8 coronal sections, which resulted in one of every eight equidistant sections (one representative section for each 240 μm) according to the rostro-caudal extent. A region of interest was drawn in each structure in Bregma −2.16 to −4.20 mm at hippocampal and hypothalamic levels and Bregma 2.28 to −0.24 mm at striatal levels, according to a rat brain atlas and cytoarchitectonic criteria (Paxinos and Watson, 2007). Phospho-H3 and BrdU-immunoreactive (-ir) nuclei that came into focus were manually counted in the SGZ and SVZ. Counting in the hypothalamus was performed in the ventromedial (VMH) and arcuate (ARC) nuclei of the hypothalamus and median eminence. Quantifications were performed using a standard optical microscope with the 40x objective (Nikon Instruments Europe B.V., Amstelveen, The Netherlands) coupled to NIS-Elements Imaging Software 3.00 (Nikon). Data are expressed as the percentage of positive cells per area (mm^2^) relative to the control group (vehicle-treated sucrose-fed rat group).

### Double immunofluorescence

The following primary antibodies were used for double immunofluorescence: rat anti-BrdU (1:500; Accurate Chemical & Scientific, Westbury, NY, USA, cat. no. OBT0030G), rabbit anti-phospho-histone H3 (1:500; Upstate, cat. no. 06-570), mouse anti-GFAP (1:500; Sigma, cat. no. G3893) and mouse anti-β3 tubulin (1:400; Promega, Madison, WI, USA). Sections were incubated overnight at 4°C with a cocktail of primary antibodies (BrdU+phospho-H3, BrdU+GFAP or BrdU+β3-tubulin). The following secondary antibodies were used when appropriate: donkey anti-rat IgG (H+L) labeled with Alexa Fluor® 488 (1:500; Molecular Probes, Invitrogen, Paisley, UK, cat. no. A21208), donkey anti-rabbit IgG (H+L) labeled with Cy™3 (1:300; Jackson Immunoresearch, West Grove, PA, USA, cat. no. 711-166-152) and donkey anti-mouse IgG (H+L) labeled with Alexa Fluor® 594 (1:500; Molecular Probes, cat. no. A21203). Sections were incubated for 2 h at room temperature with the appropriate cocktail of secondary antibodies. Sections were visualized using a confocal laser (spectral) scanning microscope (Leica TCS NT; Leica Microsystems) equipped with a 561 nm DPM laser (argon 30%) and a 63x objective (HCX PL APO CS 63.0x1.40 OIL UV). The emission filter settings were 504–545 nm for PMT2 (green) and 605–675 nm for PMT3 (red). Settings of light and brightness/contrast were adjusted using Leica LAS AF Lite imaging software. Quantification of cells showing single and double immunofluorescence was performed using several representative images of the SGZ, SVZ, and hypothalamus following the procedures described above. Data are expressed as the percentage of the total number of labeled cells that were counted per brain region.

### Statistical analysis

Statistical analysis of the results was performed using GraphPad Prism version 5.04 (GraphPad Software Inc., San Diego, CA, USA). Data are represented as the mean ± s.e.m. for at least six determinations per experimental group. Statistical analyses were performed using repeated measures ANOVA and two-way ANOVA with the two factors being time and diet (sucrose and ethanol), time and treatment (vehicle, URB597, ACEA, and JWH133) and diet and treatment, when appropriate, followed by Bonferroni *post-hoc* test for multiple comparisons. *P* < 0.05 was considered significant.

## Results

### Effects of URB597, ACEA, and JWH133 on ethanol consumption

**Figure 2:**
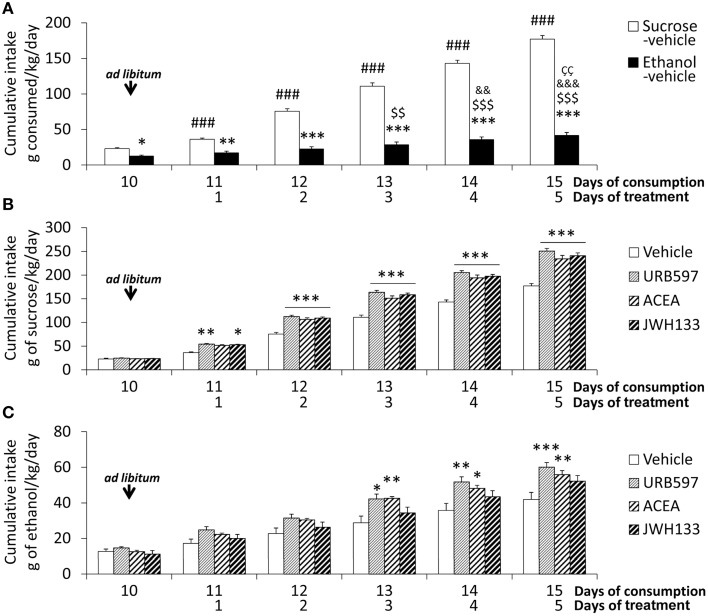
**Effects of the repeated administration of URB597 (0.3 mg/kg), ACEA (3 mg/kg), and JWH133 (0.2 mg/kg) on the cumulative ***ad libitum*** consumption of sucrose and ethanol**. **(A)** Compared consumption of sucrose and ethanol. **(B)** Treatment effects on sucrose consumption. **(C)** Treatment effects on ethanol consumption. The histograms represents the mean + s.e.m. (*n* = 6) per experimental group. Bonferroni's test following repeated measures ANOVA: ^###^*P* < 0.001 vs. the backward time points; ^$$∕$$$^*P* < 0.01/0.001 vs. day 10; ^&&∕&&&^*P* < 0.01/0.001 vs. day 11; *P* < 0.01 vs. day 12. Bonferroni's test following two-way ANOVA: ^*^P < 0.05, ^**^P < 0.01, ^***^P < 0.001 vs. the sucrose-vehicle or ethanol-vehicle groups at the same time point.

Rats were habituated to a controlled-isocaloric pair-feeding period of sucrose and alcohol diets until a stable rate of alcoholic consumption was reached (12.4 ± 1.4 g of ethanol/kg body weight/day, up to 1 week). All rats were fed *ad libitum* for 6 days after habituation (Figure 1). Bonferroni's multiple comparison test following the repeated measures ANOVA analysis indicated significant increases in the cumulative intake of sucrose diet when all time points during these 6 days were compared (^###^*P* < 0.001). However, an increase in the cumulative intake of alcohol diet was detected from day 13 onward (day 13: ^$$^*P* < 0.01; day 14 on: ^$$$^*P* < 0.001). Two-way ANOVA analysis revealed a clear diet effect when *ad libitum* sucrose and alcohol consumptions were compared [average: 30.6 ± 1.3 vs. 7.3 ± 1.1 g/kg/day; Cumulative intake: *F*_(1, 60)_ = 1104.4, *P* < 0.0001]. A time effect [*F*_(5, 60)_ = 204.8, *P* < 0.0001] and interaction between diet and time [*F*_(5, 60)_ = 97.5, *P* < 0.0001] were found, which indicates that the increased cumulative consumption of sucrose and alcohol were different over time. Bonferroni analysis revealed an increased sucrose intake compared to alcohol intake from the first day of *ad libitum* consumption (day 10: *P* < 0.05; day 11: *P* < 0.01; day 12 on: *P* < 0.001; Figure 2A).

The treatment effect on the cumulative consumption of sucrose and alcohol was evaluated from the day 11 on. Treatment effects of URB597, ACEA, and JWH133 on sucrose intake were detected [*F*_(1, 50)_ > 184.2, *P* < 0.0001]. The presence of time effects [*F*_(4, 50)_ > 390.2, *P* < 0.0001] indicated that the cumulative consumption of sucrose was different over time. An interaction between treatment and time was also observed [*F*_(4, 50)_ > 6.5, *P* < 0.0003], which indicates that the cumulative consumption of sucrose changed over time in a treatment-dependent manner. Bonferroni analyses revealed that URB597 and JWH133 increased sucrose intake from the first day of treatment (day 11 on) and ACEA increased intake from the second day (day 12 on) compared to the vehicle group (*P* < 0.001 from day 12; Figure 2B).

Treatment effects of URB597, ACEA, and JWH133 on cumulative alcohol intake were also observed [*F*_(1, 50)_ > 8.5, *P* < 0.005]. Time effects on cumulative consumption in all treated rats were detected [*F*_(4, 50)_ > 24.32, *P* < 0.0001], which indicates that the cumulative consumption of alcohol was different over time. The lack of interaction between factors suggests that the treatment similarly affected cumulative alcohol consumption over time. Bonferroni analysis demonstrated that URB597 and ACEA increased cumulative alcohol intake from the third day of treatment on compared to the vehicle group (URB597: day 13, *P* < 0.05; day 14, *P* < 0.01, day 15, *P* < 0.001; ACEA: day 13, *P* < 0.01; day 14, *P* < 0.05, day 15, *P* < 0.01). However, no statistical significance was obtained after the day-to-day analysis of JWH133 despite the overall treatment effect observed (Figure 2C).

### Effects of ethanol, URB597, ACEA, and JWH133 on hepatic transaminases, bilirubin and creatinine

We evaluated the hepatic enzymes gamma-glutamyl transpeptidase (γGT), glutamate pyruvate transaminase (GPT) and glutamic oxaloacetic transaminase (GOT), and bilirubin and creatinine in the plasma of sucrose and alcohol-fed rats to better understand the alcohol-associated toxicity and the putative protective role of cannabinoid activity (Table 1). Unbalanced levels of hepatic transaminases in plasma are generally a diagnostic indication of alcohol intoxication, and bilirubin and creatinine are important indicators of hepatic pro-oxidant ability and renal filtration, respectively.

**Table 1:** **Effects of ethanol, URB597, ACEA, and JWH133 on hepatic transaminases, bilirubin and creatinine in plasma**.

		**γGT (U/L)**	**GPT/GOT ratio**	**Bilirubin (mg/dL)**	**Creatinine (mg/dL)**
Sucrose	Vehicle	6.06±0.56	0.22±0.03	0.83±0.06	0.96±0.1
	URB597	8.13±0.79^*^	0.3±0.03	0.49±0.07^**^	0.66±0.05^*^
	ACEA	7.93±0.52^*^	0.31±0.02^*^	1.03±0.25	0.87±0.11
	JWH133	6.63±0.27	0.36±0.03^**^	0.79±0.04	0.76±0.08
Ethanol	Vehicle	7.63±0.25^*^	0.42±0.06^*^	0.9±0.04	0.99±0.02
	URB597	7.9±1.06	0.42±0.1	0.84±0.02^#^	0.57±0.04^###^
	ACEA	7.15±0.72	0.33±0.06	0.77±0.07	0.86±0.004^##^
	JWH133	6.61±0.63	0.42±0.02^*^	0.63±0.09^#^	0.68±0.07^##^

Bonferroni analysis:

* *P < 0.05*,

** *P < 0.01 vs. sucrose-vehicle group*;

# *P < 0.05 vs. ethanol-vehicle group*.

Diet effects on the GPT/GOT ratio in URB597-, ACEA-, and JWH133-treated rats were found [URB597: *F*_(1, 20)_ = 5.9, *P* = 0.024; ACEA: *F*_(1, 20)_ = 5.1, *P* = 0.035; JWH133: *F*_(1, 20)_ = 9.5, *P* = 0.005]. We only detected an alcohol effect on the plasma levels of bilirubin in URB597-treated rats [*F*_(1, 20)_ = 13.5, *P* = 0.001]. No alcohol effect was observed on the plasma levels of γGT or creatinine. We detected treatment effects of URB597 and JWH133 on the plasma levels of bilirubin [URB597: *F*_(1, 20)_ = 13.3, *P* = 0.0016; JWH133: *F*_(1, 20)_ = 5.5, *P* = 0.028]. We also observed treatment effects of URB597 and JWH133 on plasma creatinine levels [URB597: *F*_(1, 20)_= 30.4, *P* < 0.0001; JWH133: *F*_(1, 20)_ = 10.4, *P* = 0.004]. The plasma levels of γGT and GPT/GOT ratio did not exhibit any treatment effects. Interactions between diet and treatment were specifically observed in the γGT levels of ACEA-treated rats [*F*_(1, 20)_ = 4.6, *P* = 0.043] and the bilirubin levels of URB597-treated rats [*F*_(1, 20)_ = 6.4, *P* = 0.019].

Bonferroni analyses indicated that alcohol increased the plasma levels of γGT and the GPT/GOT ratio (^*^P < 0.05; Table 1). URB597 and ACEA increased the plasma levels of γGT (^*^P < 0.05), ACEA and JWH133 increased the GPT/GOT ratio (^*^P < 0.05, ^**^P < 0.01, respectively), and URB597 decreased the plasma levels of bilirubin and creatinine (^**^P < 0.01, ^*^P < 0.05, respectively) in sucrose-fed rats (Table 1). URB597 and JWH133 decreased the plasma levels of bilirubin (^#^*P* < 0.05), and URB597, ACEA, and JWH133 decreased plasma creatinine levels (^###^*P* < 0.001, ^##^*P* < 0.01, ^##^*P* < 0.01, respectively) in ethanol-fed rats (Table 1).

### Effects of ethanol, URB597, ACEA, and JWH133 on NPC proliferation

We evaluated the percentage of neural proliferating cells that expressed the mitotic phosphorylation of (Ser-10)-histone 3 (phospho-H3+) and contained the replicating cell DNA marker 5-bromo-2′-deoxyuridine (BrdU+) in the main neurogenic zones of the adult brain (SGZ, SVZ, and hypothalamus).

No alcohol effects on the number of phospho-H3+ cells were detected in the SGZ or hypothalamus. No alcohol effects on the number of BrdU+ cells in SVZ of treated rats were detected. However, we observed alcohol effects on the number of BrdU+ cells in the SGZ of URB597-, ACEA-, and JWH133-treated rats [*F*_(1, 32)_ > 11.64, *P* < 0.0018] and the hypothalamus of URB597- and JWH133-treated rats [*F*_(1, 32)_ > 11.83, *P* < 0.0016]. An alcohol effect on the number of phospho-H3+ cells in ACEA-treated rats was specifically observed in the SVZ [*F*_(1, 32)_ = 12.38, *P* = 0.001] (Table 2).

**Table 2:** **Summary of the effects of diet, treatment and interaction observed on NPC proliferation after two-way ANOVA analysis**.

	**SGZ**		**SVZ**		**Hypothalamus**	
	**Phospho-H3**	**BrdU**	**Phospho-H3**	**BrdU**	**Phospho-H3**	**BrdU**
**URB597**						
Diet	ns	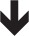 ^**^	ns	ns	ns	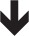 ^**^
Treatment	ns	ns	ns	ns	ns	ns
Interaction	ns	ns	^*^	^*^	ns	ns
**ACEA**						
Diet	ns	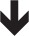 ^***^	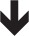 ^**^	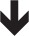 ^*^	ns	ns
Treatment	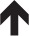 ^***^	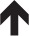 ^**^	ns	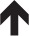 ^***^	ns	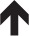 ^***^
Interaction	ns	ns	^*^	ns	ns	ns
**JWH133**						
Diet	ns	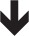 ^**^	ns	ns	ns	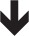 ^***^
Treatment	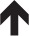 ^***^	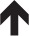 ^***^	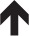 ^***^	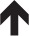 ^***^	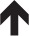 ^*^	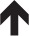 ^**^
Interaction	ns	ns	ns	^**^	ns	ns

Two-way ANOVA:

* *P < 0.05*;

** *P < 0.01*;

*** *P < 0.001; ns, no significance. Direction of the change: 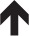, increased levels; 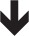, decreased levels*.

Treatment effects of ACEA and JWH133, but not URB597, on the number of phospho-H3+ and BrdU+ cells in the SGZ were observed [ACEA: *F*_(1, 32)_ > 12.04, *P* < 0.0015; JWH133: *F*_(1, 32)_ > 22.53, *P* < 0.0001] (Table 2). We specifically found effects of JWH133 on the number of phospho-H3+ and BrdU+ cells in the SVZ and hypothalamus [SVZ: *F*_(1, 32)_ > 25.54, *P* < 0.0001; hypothalamus: *F*_(1, 32)_ > 5.27, *P* < 0.02]. ACEA effects on the number of BrdU+ cells were also observed in these areas [SVZ: *F*_(1, 32)_ = 4.62, *P* = 0.039; hypothalamus: *F*_(1, 32)_ = 14.63, *P* = 0.0006]. These data suggest similar effects of the selective activation of CB1 and CB2 receptors on cell proliferation in the SGZ, SVZ, and hypothalamus in a diet-independent manner (Table 2).

We did not find interactions between factors (diet and treatment) when we analyzed the number of phospho-H3+ and BrdU+ cells in the SGZ and hypothalamus of treated rats. Interactions were detected in the SVZ when we statistically analyzed the number of phospho-H3+ cells in URB597- and ACEA-treated rats [*F*_(1, 32)_ > 5.22, *P* < 0.029] and the number of BrdU+ cells in URB597-treated rats [*F*_(1, 32)_ = 4.97, *P* = 0.033]. These results indicated that treatment primarily affected SVZ cell proliferation in a diet-dependent manner (Table 2).

**Figure 3:**
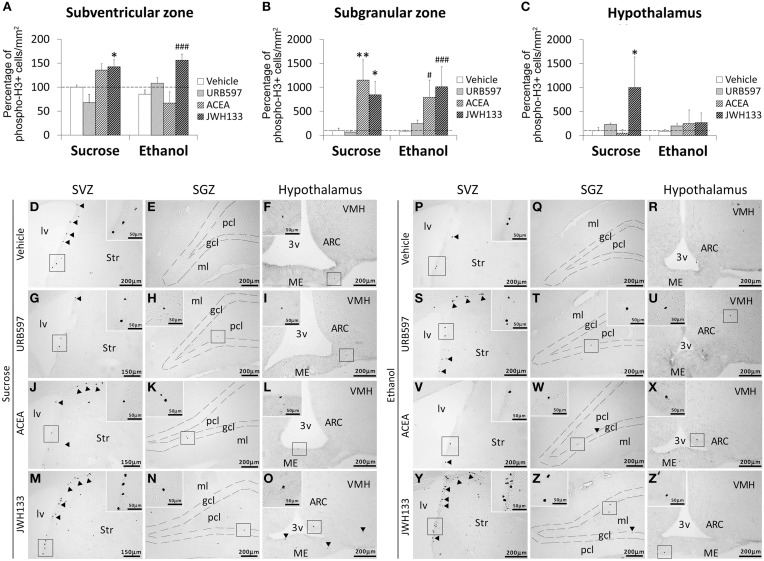
**Effects of the repeated administration of URB597 (0.3 mg/kg), ACEA (3 mg/kg), and JWH133 (0.2 mg/kg) on cells that expressed the mitotic Ser-10 phosphorylation of histone-3 (phospho-H3+) in the SVZ (A), SGZ (B), and hypothalamus (C) of adult rats with sucrose and alcohol consumption**. The data were expressed as the percentage of positive cells per area (mm^2^) relative to the control group (vehicle-treated sucrose-fed group). The histograms represents the mean + s.e.m. (*n* = 6). Bonferroni's test: ^*^P < 0.05, ^**^P < 0.01 vs. sucrose-vehicle group; ^#^*P* < 0.05, ^###^*P* < 0.001 vs. ethanol-vehicle group. **(D–Z')** Representative microphotographs showing low and high (insets) magnification views of cells expressing phospho-H3 (arrowheads) in the SGZ, SVZ, and hypothalamus of vehicle, URB597, ACEA, and JWH133-treated rats fed with sucrose (left panel) and ethanol (right panel) diets. Scale bars are included in each image.

Bonferroni analyses for multiple comparisons indicated that ethanol did not produce any changes in the percentage of phospho-H3+ cells in the SVZ (^*^P < 0.05), SGZ (^***^P < 0.001) or hypothalamus (^*^P < 0.05; Figures 3A–C). JWH133 increased the percentage of phospho-H3+ in the SVZ of sucrose- (^*^P < 0.05) and ethanol-fed rats (^###^*P* < 0.001; Figure 3A). ACEA and JWH133 increased the percentage of phospho-H3+ cells in the SGZ of sucrose- (^**^P < 0.01, ^*^P < 0.05, respectively) and ethanol-fed rats (^#^*P* < 0.05, ^###^*P* < 0.001, respectively; Figure 3B). JWH133 increased the percentage of phospho-H3+ in the hypothalamus of sucrose-fed rats (*P < 0.05; Figure 3C). Representative images of the labeling in the SVZ, SGZ, and hypothalamus of each experimental group are shown in Figures 3D–Z′.

**Figure 4:**
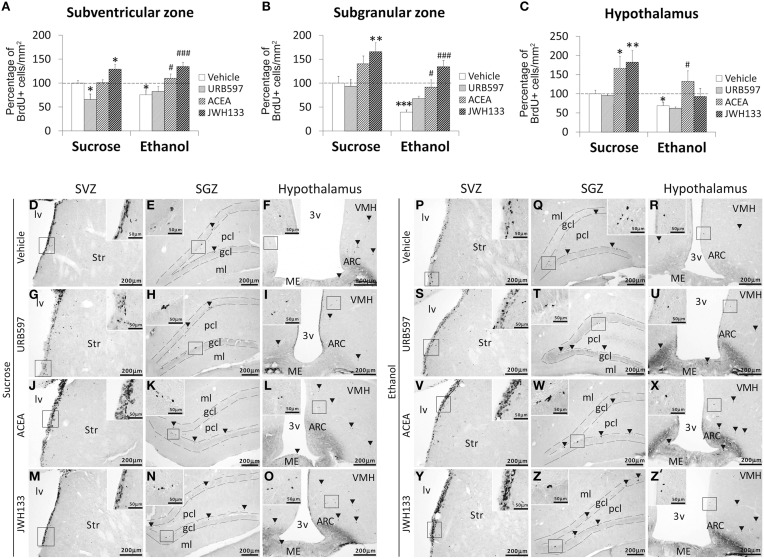
**Effects of the repeated administration of URB597 (0.3 mg/kg), ACEA (3 mg/kg), and JWH133 (0.2 mg/kg) on cells that contained the replicating cell DNA marker 5-bromo-2′-deoxyuridine (BrdU+) in the SVZ (A), SGZ (B), and hypothalamus (C) of adult rats with sucrose and alcohol consumption**. The data were expressed as the percentage of positive cells per area (mm^2^) relative to the control group (vehicle-treated sucrose-fed group). The histograms represents the mean + s.e.m. (*n* = 6). Bonferroni's test: ^*^P < 0.05, ^**^P < 0.01, ^***^P < 0.001 vs. sucrose-vehicle group; ^#^*P* < 0.05, ^###^*P* < 0.001 vs. ethanol-vehicle group. **(D–Z**′**)** Representative microphotographs showing low and high (insets) magnification views of the typical clustering of newborn cells containing the BrdU labeling (arrowheads) in the SGZ, SVZ, and hypothalamus of vehicle, URB597, ACEA, and JWH133-treated rats fed with sucrose (left panel) and ethanol (right panel) diets. Scale bars are included in each image.

Bonferroni analyses for multiple comparisons indicated that ethanol induced a decrease in the percentage of BrdU+ cells in the SVZ (^*^P < 0.05), SGZ (^***^P < 0.001), and hypothalamus (^*^P < 0.05; Figures 4A–C). JWH133 increased the percentage of BrdU+ cells in the SVZ and SGZ of sucrose- and ethanol-fed rats (Figures 4A,B). ACEA and JWH133 increased the percentage of BrdU+ cells in the hypothalamus of sucrose-fed rats (Figure 4C). ACEA increased the percentage of BrdU+ cells in the SVZ, SGZ, and hypothalamus of ethanol-fed rats (^#^*P* < 0.05; Figures 4A–C). Representative images of the labeling in the SVZ, SGZ, and hypothalamus of each experimental group are shown in Figures 4D–Z′.

### Phenotyping of neural proliferating cells

**Figure 5:**
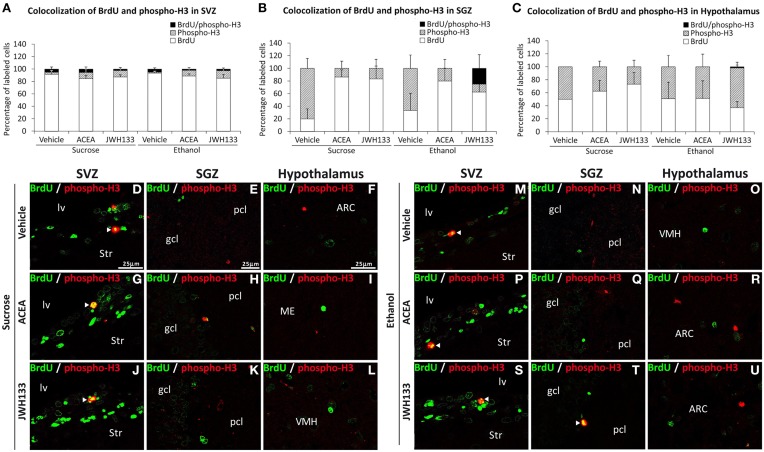
**Quantification of the proportion of cells labeled with BrdU that expressed phospho-H3 in the SVZ (A), SGZ (B), and hypothalamus (C) of vehicle, ACEA and JWH133-treated rats fed with sucrose and ethanol diets**. Bars represent the percentage of labeled cells in each experimental group. **(D–U)** BrdU and phospho-H3 co-expression in the rat brain. High-resolution confocal laser scanning photomicrographs showing the labeling of BrdU (green) and phospho-H3 (red) in the SVZ, SGZ, and hypothalamus of vehicle, ACEA and JWH133-treated rats fed with sucrose (left panel) and ethanol (right panel) diets. The arrowheads indicate co-expression. Scale bars are included in the left-upper images.

We evaluated the percentage of BrdU+ cells showing a precursor (GFAP or phospho-H3 expression) or mature neuronal (β3-tubulin) phenotype to characterize the neural proliferating cells in the main neurogenic zones of ACEA- and JWH133-treated rats. We detected a very low number and a lack of BrdU+ cells expressing the mitotic marker phospho-H3 in the SVZ and hypothalamus, respectively (Figures 5A,C). In contrast, alcohol-exposed rats treated with JWH133, but not the other treatments, exhibited BrdU+ cells that expressed phospho-H3 in the SGZ (Figure 5B). Representative images of BrdU and phospho-H3 labeling in the SVZ, SGZ, and hypothalamus of each experimental group are shown in Figures 5D–U.

**Figure 6:**
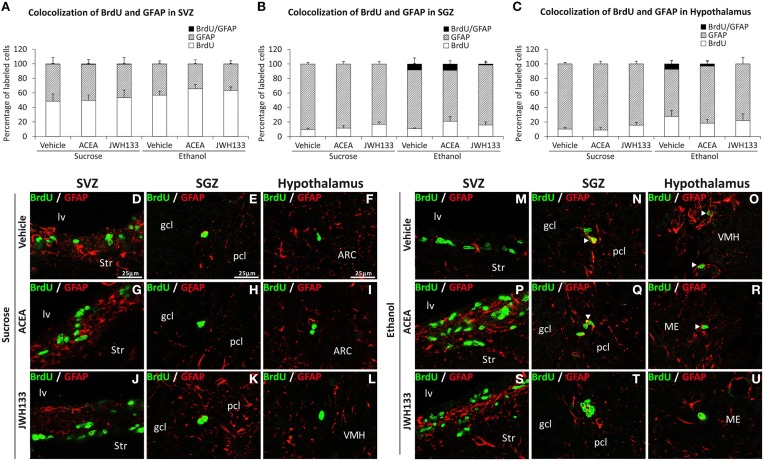
**Quantification of the proportion of cells labeled with BrdU that expressed GFAP in the SVZ (A), SGZ (B), and hypothalamus (C) of vehicle, ACEA and JWH133-treated rats fed with sucrose and ethanol diets**. Bars represent the percentage of labeled cells in each experimental group. **(D–U)** BrdU and GFAP co-expression in the rat brain. High-resolution confocal laser scanning photomicrographs showing the labeling of BrdU (green) and GFAP (red) in the SVZ, SGZ, and hypothalamus of vehicle, ACEA and JWH133-treated rats fed with sucrose (left panel) and ethanol (right panel) diets. The arrowheads indicate co-expression. Scale bars are included in the left-upper images.

The SGZ and hypothalamus of the rats exposed to alcohol exhibited BrdU+ cells that expressed the radial/astrocyte precursor GFAP, which was not detected when alcohol-exposed rats were treated with JWH133 (Figures 6B,C). No co-expression of BrdU and GFAP in the SVZ was observed (Figure 6A). Representative images of the BrdU and GFAP labeling in the SVZ, SGZ, and hypothalamus of each experimental group are shown in Figures 6D–U.

**Figure 7:**
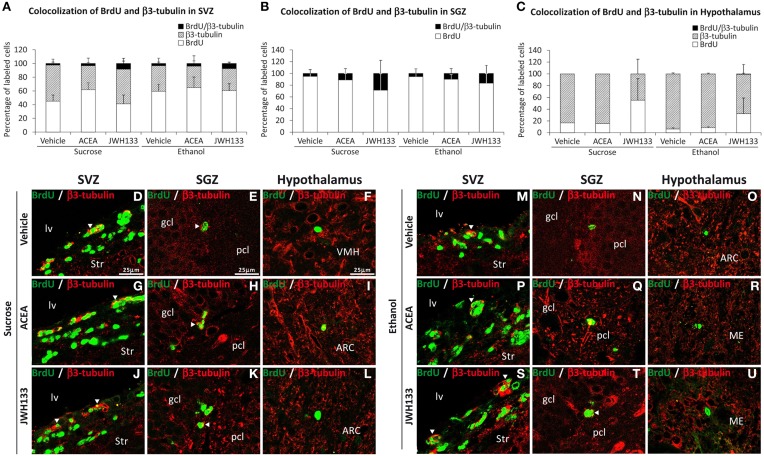
**Quantification of the proportion of cells labeled with BrdU that expressed β3-tubulin in the SVZ (A), SGZ (B), and hypothalamus (C) of vehicle, ACEA and JWH133-treated rats fed with sucrose and ethanol diets**. Bars represent the percentage of labeled cells in each experimental group. **(D–U)** BrdU and β3-tubulin co-expression in the rat brain. High-resolution confocal laser scanning photomicrographs showing the labeling of BrdU (green) and β3-tubulin (red) in the SVZ, SGZ, and hypothalamus of vehicle, ACEA and JWH133-treated rats fed with sucrose (left panel) and ethanol (right panel) diets. The arrowheads indicate co-expression. Scale bars are included in the left-upper images.

All rat groups treated with JWH133 exhibited an increased number of BrdU+ cells that expressed the neuron-specific β3-tubulin in the SVZ and SGZ (Figures 7A,B). No co-expression of BrdU and β3-tubulin in the hypothalamus was observed (Figure 7C). Representative images of BrdU and β3-tubulin labeling in the SVZ, SGZ, and hypothalamus of each experimental group are shown in Figures 7D–U.

## Discussion

**Figure 8:**
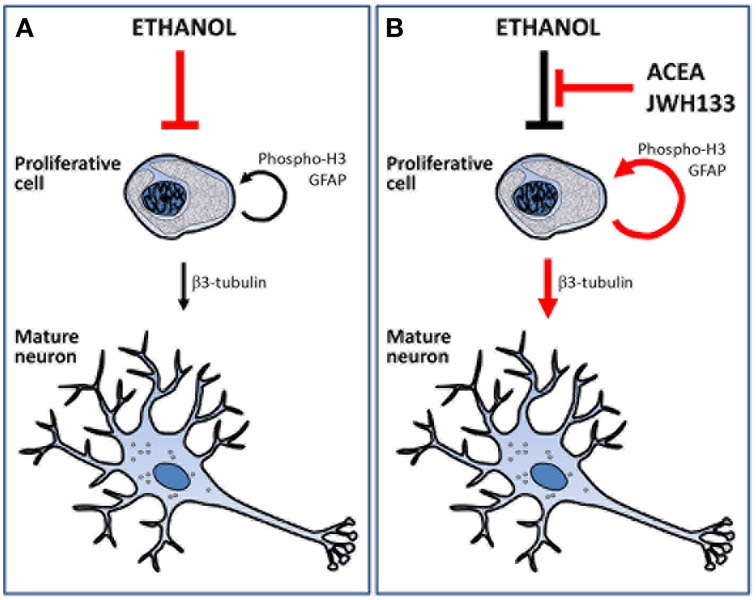
**Schematic representation that summarize the main effects of alcohol, ACEA and JWH133 on cell proliferation and neuronal maturation**. **(A)** Ethanol reduced the number of proliferative cells and, as a consequence, the number of cell showing neuronal phenotype. **(B)** ACEA and JWH can block the deleterious effects of alcohol as they increased the number of proliferative cells and the putative mature neurons.

The present study demonstrated that URB597 and ACEA increased alcohol consumption likely via CB_1_ receptor activation, but the selective stimulation of CB_1_ and CB_2_ receptors using ACEA and JWH133 actively counteracted the alcohol-induced decrease in NPC proliferation in the brain of adult rats with a forced consumption of alcohol (Figure 8). Data analyses revealed treatment effects on cell proliferation in a neurogenic region-dependent manner. Therefore, we reached the following conclusions: (1) ACEA and JWH133 induced an increase in NPC proliferation (phospho-H3+ and BrdU+ cells) in the SGZ of the alcohol-exposed rats, which indicates a non-selective cannabinoid receptor-dependent stimulation of SGZ cell proliferation, and (2) JWH133 also produced a specific increase in NPC proliferation (phospho-H3+ and BrdU+ cells) in the SVZ of alcohol-exposed rats, which indicates a selective CB_2_ receptor activation of SVZ cell proliferation. The increased cell proliferation after CB_2_ receptor activation may be related to an improvement in adult neurogenesis because JWH133 induced numerous BrdU+ cells that expressed neuron-specific β3-tubulin in the SGZ and SVZ. The effects of JWH133 on neurogenesis must be contextualized with the lack of significant increase in alcohol consumption and, as a probable consequence, the reduced concentration of bilirubin and creatinine in the plasma of alcohol-exposed rats, which indicates an amelioration of the hepatic anti-oxidant activity and renal filtration, respectively. This information suggests that CB_2_ receptor activation by JWH133 is the most suitable strategy to counteract the toxicity effects of alcohol on neurogenesis in the two main neurogenic zones (SGZ and SVZ) of the adult brain. However, the increase in BrdU+ cells, but not phospho-H3+ cells, in the SVZ and hypothalamus by the selective CB_1_ receptor agonist ACEA suggests a less potent recovery of cell proliferation in alcohol-exposed rat brain. Selective stimulation of the cannabinoid CB_1_ and CB_2_ receptors also increased NPC proliferation in a neurogenic region-dependent manner in rats fed a sucrose liquid diet. Notably, the most consistent results also highlighted the effects of CB_2_ receptor activation on the brain of sucrose-fed rats because JWH133 increased NPC proliferation (phospho-H3+ and BrdU+ cells) in the SGZ, SVZ, and hypothalamus.

Our results are consistent with previous studies that demonstrated the following results: (1) an *in vitro* promotion of the neurosphere generation and an *in vivo* stimulation of hippocampal NPC proliferation by WIN 55,212-2 and AEA (Aguado et al., 2005); (2) an increased number of Ki67+ cells in the SVZ of mice treated with JWH133 and WIN 55,212-2; and (3) a decreased number of Ki67+ cells in the SVZ of mice treated with the CB_2_ receptor antagonists AM630 and JTE-907 (Goncalves et al., 2008). Our results provide inconclusive results on the URB597 effect on NPC proliferation in the SGZ and SVZ, which is consistent with previous studies. An increased proliferation of neural progenitors (BrdU+ and/or Ki67+ cells) was described in the hippocampus of *FAAH*^−∕−^ mice, the SVZ of URB597-treated mice and the neurospheres treated *in vitro* by URB597 (3 nM; Aguado et al., 2005; Goncalves et al., 2008). In contrast, a recent study by Rivera et al. (2015) demonstrated that acute treatment with URB597 (0.3 mg/kg) increased the number of phospho-H3+ and BrdU+ cells specifically in the SVZ of the adult rat brain, but no change was detected in the SGZ. These authors demonstrated that the repeated administration of URB597 specifically reduced NPC proliferation in the SGZ of the adult rat brain (Rivera et al., 2015). URB597 at a dose of 0.3 mg/kg maximally blocked FAAH activity and increased plasma and brain levels of AEA in alcohol-exposed rats (see Figure S1), but the same neurogenic effect was not produced in the SGZ using ACEA or JWH133. Therefore, we postulated that the differential effects of URB597 on NPC proliferation may be a consequence of the conjunction of multiple factors, such as the different catalytic properties of FAAH, depending on substrate specificity (e.g., anandamide, oleoylethanolamide, and palmithylethanolamide), the cell types that express FAAH (e.g., FAAH is highly expressed in the hippocampus and lowly expressed in the hypothalamus), and the physiopathological conditions of the experimental model (Sun et al., 2005; Ohno-Shosaku and Kano, 2014).

Our results demonstrated similar effects of URB597 and ACEA on sucrose and alcohol consumption, which suggests an indiscriminate consumption response via CB_1_ receptor activation, but JWH133 did not demonstrate a specific effect on ethanol intake. In contrast to the increased sucrose intake after CB_2_ receptor stimulation, our results demonstrated a very low influence of JWH133 (and thus cannabinoid CB_2_ receptors) in forced alcohol consumption. These results agree with the contradictory responses of cannabinoid signaling in the promotion of voluntary alcohol consumption and the low motor performance-induced blockade of operant alcohol self-administration (Serrano and Parsons, 2011). The following previous results were described: (1) WIN 55,212-2 and CP 55,940 promote voluntary ethanol intake in sP rats (Colombo et al., 2002); (2) ACEA, WIN 55,212-2, AM404, and URB597 reduce alcohol self-administration, which likely indicates a compensation/substitution of the reinforcing effects of ethanol (Cippitelli et al., 2007); and 3) *CB*_1_^−∕−^ mice consumed less alcohol, and the CB_1_ receptor antagonist SR141716A blocked the dopamine-releasing effects of alcohol and reduced operant alcohol self-administration (Hungund et al., 2003; Crabbe et al., 2006; Hansson et al., 2007). These studies contrast with other studies that showed that CB_2_ receptor agonists reduced alcohol preference in mice (Vasiljevik et al., 2013; Al Mansouri et al., 2014).

The increased concentration of hepatic transaminases in the plasma of alcohol-exposed rats should be associated with an alcohol-induced decrease in BrdU+ cells, but not phospho-H3+ cells, in the three neurogenic zones analyzed (SGZ, SVZ, and hypothalamus). We proposed three non-exclusive hypothesis to explain the differential effects of alcohol on phospho-H3+ and BrdU+ cells: (1) the basal low rate of phospho-H3+ cell production could hide the deleterious effect of ethanol; (2) the average ethanol intake of 7.3 ± 1.1 g/kg/day for the last 6 days did not produce a relevant deleterious effect on the rate of phospho-H3+ cells; and (3) BrdU+ cells that represent neuroblasts and young immature neurons exhibited a higher vulnerability to alcohol toxicity and a higher risk of cell death (Deng et al., 2010).

In summary, the present study demonstrated that selective CB_2_ receptor agonist JWH133 counteracted the deleterious effect of alcohol on NPC proliferation in the SGZ and SVZ, but the selective CB_1_ receptor agonist ACEA only reversed the impaired NPC proliferation in the SGZ. ACEA increased alcohol consumption, but JWH133 did not, which suggests that the activation of CB_2_ receptors is of potential clinical interest for the risk of neural damage in alcohol dependence. Further studies are required to assess whether the effects of selective cannabinoid CB_1_/CB_2_ stimulation on neurogenesis can induce the recovery of hippocampal-dependent tasks in alcoholic patients.

## Author contributions

All authors had full access to all data in the study and take responsibility for the integrity of the data and the accuracy of the data analyses. Study concept and design: FR, JS. Acquisition of data: PR, EB, LB, FA, AV, LR. Analysis and interpretation of data: PR, EB, FP, AS, FR, JS. Drafting of the manuscript: FR, JS. Critical revision of the manuscript for important intellectual content, obtained funding and study supervision: BL, FR, JS.

### Conflict of interest statement

The authors declare that the research was conducted in the absence of any commercial or financial relationships that could be construed as a potential conflict of interest.

## Supplementary material

Figure S1 **Effect of URB597 on AEA levels in the plasma and the brain regions striatum, hippocampus and hypothalamus of ethanol-fed rats**. Bonferroni's test: ^*^*P* < 0.05, ^**^*P* < 0.01, ^***^*P* < 0.001 vs. sucrose-fed rats; ^###^*P* < 0.001 vs. ethanol-fed rats.

## Abbreviations

3v: third ventricle
ACEA: arachidonyl-2′-chloroethylamide
ARC: arcuate nucleus of hypothalamus
BrdU: 5-bromo-2′-deoxyuridine
CB_1_/CB_2_ receptors: cannabinoid receptor types 1 and 2
gcl: granular cell layer
FAAH: fatty acid amide hydrolase
γGT: gamma-glutamyl transferase
GOT: glutamic-oxaloacetic transaminase
GPT: glutamic-pyruvic transaminase
HC: hippocampus
HT: hypothalamus
lv: lateral ventricle
ME: median eminence
ml: molecular layer
pcl: polymorphic cell layer
SGZ: subgranular zone of dentate gyrus
Str: striatum
SVZ: subventricular zone of the lateral ventricles
URB597: cyclohexyl carbamic acid 3′-carbamoyl-biphenyl-3-yl ester
VMH: ventromedial nucleus of hypothalamus.

**Chemical compounds studied in this article:**
URB597: (PubChem CID: 1383884)
ACEA: (PubChem CID: 5311006)
JWH133: (PubChem CID: 6918505).
