# Supplementary figures and images for: Pharmacological activation of CB2 receptors counteracts the deleterious effect of ethanol on cell proliferation in the main neurogenic zones of the adult rat brain
Source: Front Cell Neurosci. 2015 Sep 29;9:379. doi: 10.3389/fncel.2015.00379 (PMC4587308; doi:10.3389/fncel.2015.00379)

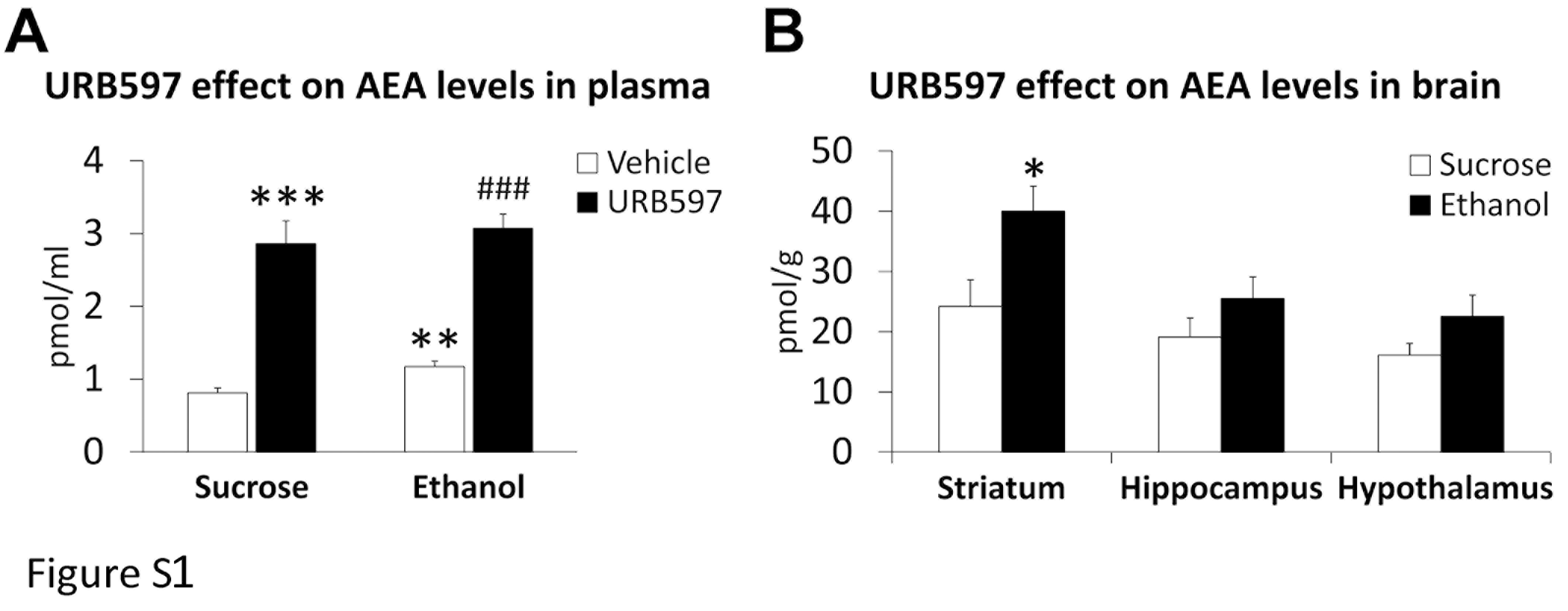

Supplement: Figure S1 — Effect of URB597 on AEA levels in the plasma and the brain regions striatum, hippocampus and hypothalamus of ethanol-fed rats. Bonferroni's test: *P < 0.05, **P < 0.01, ***P < 0.001 vs. sucrose-fed rats; ###P < 0.001 vs. ethanol-fed rats. [file Image1.TIF]
