# Supplementary material for: Pharmacological activation of CB2 receptors counteracts the deleterious effect of ethanol on cell proliferation in the main neurogenic zones of the adult rat brain
Source: Front Cell Neurosci. 2015 Sep 29;9:379. doi: 10.3389/fncel.2015.00379 (PMC4587308; doi:10.3389/fncel.2015.00379)
Supplement: Supplementary file 2 [file DataSheet1.DOC]

**SUPPLEMENTARY TEXT**

**RESULTS**

**Effects of ethanol and URB597 on the plasma and brain levels of anandamide**

We analyzed the plasma and brain levels of AEA in rats fed with sucrose and alcoholic diets two hours after the last administration of URB597 (Fig. S1).

A treatment effect of URB597 on the plasma levels of AEA was observed (*F*_1,20_=102.21, *P*<0.0001). No diet effect and interaction between diet and treatment were evidenced on the plasma levels of AEA. Bonferroni analysis indicated that alcohol consumption increased the plasma levels of AEA (***P*<0.01) (Fig. S1A). As we expected, increased levels of AEA were observed in the plasma of URB597-treated rats fed with both sucrose (****P*<0.001) and ethanol (^###^*P*<0.001) liquid diets (Fig. S1A).

A diet effect on the brain levels of AEA was observed in the rats treated with URB597 (*F*_1,30_=10.62, *P*=0.002), which indicates increased AEA levels in the brain of URB-treated rats. Regarding striatum, hippocampus and hypothalamus, a brain region effect on the AEA levels was also found (*F*_2,30_=6.95, *P*=0.003), which indicates differences of the AEA levels in a brain region-dependent manner. No interaction between diet and brain regions was detected. Bonferroni analysis indicated that striatal levels of AEA were specifically significant in alcoholic rats treated with URB597 (**P*<0.05) (Fig. S1B).

**REFERENCE**

Lomazzo E, Bindila L, Remmers F, Lerner R, Schwitter C, Hoheisel U, Lutz B (2015) Therapeutic potential of inhibitors of endocannabinoid degradation for the treatment of stress-related hyperalgesia in an animal model of chronic pain. Neuropsychopharmacology 40:488-501. doi: 10.1038/npp.2014.198.
